# Deciding on the Starting Number of Classes of a Latent Class Tree

**DOI:** 10.1177/0081175018780170

**Published:** 2018-06-21

**Authors:** Mattis van den Bergh, Geert H. van Kollenburg, Jeroen K. Vermunt

**Affiliations:** * Tilburg University, Tilburg, the Netherlands; † Radboud University, Nijmegen, the Netherlands

**Keywords:** latent class analysis, classification trees, mixture models, categorical data analysis, latent class trees, model selection

## Abstract

In recent studies, latent class tree (LCT) modeling has been proposed as a convenient alternative to standard latent class (LC) analysis. Instead of using an estimation method in which all classes are formed simultaneously given the specified number of classes, in LCT analysis a hierarchical structure of mutually linked classes is obtained by sequentially splitting classes into two subclasses. The resulting tree structure gives a clear insight into how the classes are formed and how solutions with different numbers of classes are substantively linked to one another. A limitation of the current LCT modeling approach is that it allows only for binary splits, which in certain situations may be too restrictive. Especially at the root node of the tree, where an initial set of classes is created based on the most dominant associations present in the data, it may make sense to use a model with more than two classes. In this article, we propose a modification of the LCT approach that allows for a nonbinary split at the root node, and we provide methods to determine the appropriate number of classes in this first split, based either on theoretical grounds or on a relative improvement of fit measure. This novel approach also can be seen as a hybrid of a standard LC model and a binary LCT model, in which an initial, oversimplified but interpretable model is refined using an LCT approach. Furthermore, we show how to apply an LCT model when a nonstandard LC model is required. These new approaches are illustrated using two empirical applications: one on social capital and the other on (post)materialism.

## 1. Introduction

Latent class (LC) modeling has become a popular tool for clustering respondents into homogeneous subgroups based on their responses on a set of categorical variables (Clogg 1995; Goodman 1974; Hagenaars 1990; Lazarsfeld and Henry 1968; Magidson and Vermunt 2004; McCutcheon 1987; Vermunt and Magidson 2002). LC models have been applied to the investigation of a variety of subjects—for example, risk behavior such as gambling (Studer et al. 2015) and suicide attempts (Thullen, Taliaferro, and Muehlenkamp 2015), social constructs such as social class (Savage et al. 2013) and social support (Santos et al. 2015), and cognitive constructs such as rule assessment (Jansen and van der Maas 1997) and cognitive control (Van Hulst, De Zeeuw, and Durston 2015).

A crucial part of doing an LC analysis is the decision on the required number of classes. In a confirmatory setting, the number of classes may be based on a priori knowledge, though the specified LC model may not fit due to, for instance, the presence of subclasses or other kinds of mechanisms causing violations of the local independence assumption. In such situations, it may make sense to relax the local independence assumption, as suggested by Oberski (2016), among others.

In an exploratory setting, we will typically aim not at finding the “true” number of clusters but instead will look for a clustering that describes the data reasonably well and is moreover easy to interpret. To achieve this goal, researchers estimate models with different numbers of classes and select the model that performs best according to some fit measure—for example, according to the Akaike information criterion (AIC) or the Bayesian information criterion (BIC). While AIC and BIC penalize model complexity and thus prefer models with fewer classes, when applying LC models to data sets that are (very) large in terms of number of cases and/or number of variables, we will often end up with a model having a large number of classes. Some of these classes may differ from one another in very specific and possibly less interesting ways, making their distinction hard to interpret substantively. Moreover, different model selection measures will typically point at different best models in terms of the number of classes. In such situations, researchers can no longer rely on purely statistical criteria but will instead need to inspect solutions with a different number of classes and probably opt for the model that best fits their substantive goals (e.g., Hadiwijaya et al. 2015; Oser, Hooghe, and Marien 2013; Spycher et al. 2008; Sullivan, Kessler, and Kendler 1998). It will be clear that such an approach may be somewhat problematic since different researchers may come up with different final models when analyzing exactly the same data without being able to substantively relate the different results.

To overcome the above-mentioned problems associated with LC analysis applications with large data sets, van den Bergh, Schmittmann, and Vermunt (2017) proposed an alternative way of performing an LC analysis, which they called latent class tree (LCT) analysis. Their approach involves performing a divisive hierarchical cluster analysis using an algorithm developed by Van der Palm, Van der Ark, and Vermunt (2016) for density estimation with a large number of categorical variables. The main advantage of the LCT modeling approach is that it shows how models with different numbers of classes are linked to one another; for instance, a model with six classes is a model with five classes in which one of the classes split into two parts. When applying an LCT, the model selection problem is reduced to deciding whether a particular split should be accepted—should be a *yes* or a *no.* As in a standard LC analysis, this can be decided according to fit measures but also according to whether a split is meaningful based on content.

**Figure 1.**
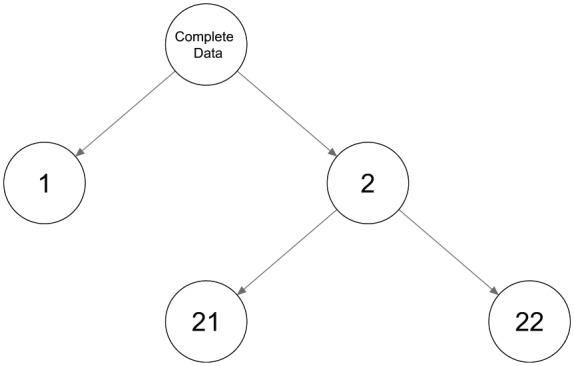
An example of a tree structure with two binary splits.

As the name suggests, the LCT method yields a tree structure (see Figure 1 for an example), which at the top contains a root node that serves as a “parent” node of two “child” nodes. At the next level of the tree, these child nodes become parent nodes and produce possibly their own child nodes, and so on. More specifically, the algorithm used to construct an LCT works as follows: Initally, a one- and two-class model is estimated for the root node—that is, using the original data set. If the two-class model is preferred according to the model selection criterion used, then two child nodes are created. For each of the two child nodes a new data set is constructed, which contains the posterior membership probabilities for the class concerned as case weight. Subsequently, each new child node is treated as a parent, and we can check whether a two-class model provides a better fit than a one-class model on the corresponding weighted data set. This stepwise procedure continues until no additional nodes are split up.

The sequential LCT algorithm yields child classes that are subclasses of a parent class, which implies that interpretation can take place at any level of the tree. That is, after labeling the classes formed at the root of the tree, the classes formed at the next level of the tree will be labeled conditionally on the labeling of their parent classes. This makes it much easier to interpret LC solutions with more than a few classes. Moreover, the fact that the classes are hierarchically linked makes it possible to decide on the number of classes based on substantive interpretation of the splits; if certain splits are not interesting or relevant for the research question at hand, the child classes of a split can be substituted for their parent class. Hierarchical tree structures similar to those obtained with an LCT analysis are very practical as clustering procedures because clustering solutions at different levels of a tree allow different granularity to be extracted during the data analysis, making them ideal for exploration (Ghattas, Michel, and Boyer 2017; Zhao, Karypis, and Fayyad 2005).

**Figure 2.**
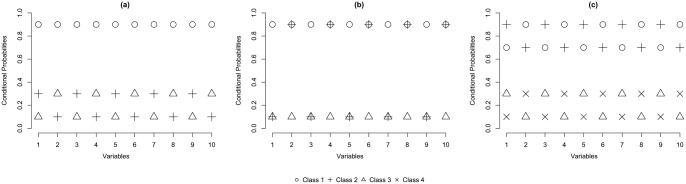
Two examples of three classes and one of four classes.

Limiting the number of classes with binary splits in an LCT is a practical but dangerous restriction. As an illustration of this problem, Figure 2 presents three examples of possible LC configurations: two with three classes and one with four classes. Figure 2(a), the first configuration of three classes, shows two fairly similar classes (classes 2 and 3), while class 1 is quite distinct from these two. This is a situation in which a tree with binary splits is expected to perform well. In the first binary split, class 1 will be separated from classes 2 and 3, where the class combining the latter two will have response probabilities close to 0.2 (the average of these two classes). The binary split at the next level will detect the differences between classes 2 and 3. Hence, binary splits do not cause any problems with this setup, and an example of the resulting tree structure is shown by Figure 1, where classes 2 and 3 are defined as 21 and 22 in the tree structure.

**Figure 3.**
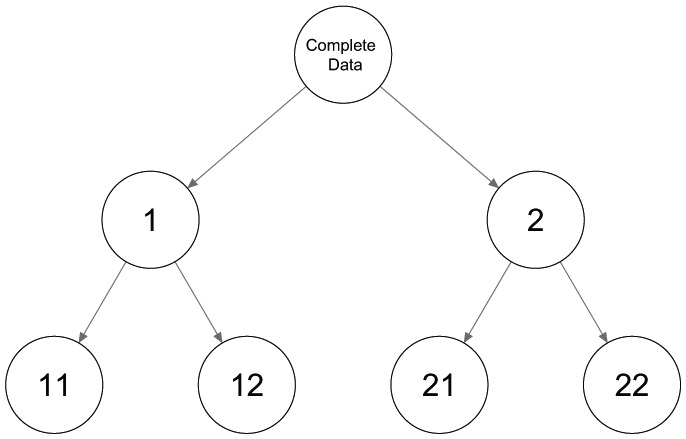
Tree structures with three binary splits.

**Figure 4.**
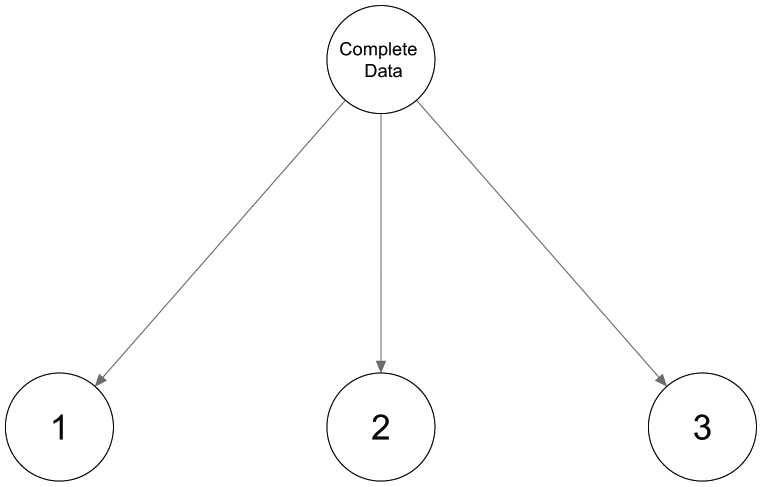
A three-class latent class model.

The second configuration of three classes in Figure 2(b) shows three rather distinct classes. The first binary split will be based mainly on most dissimilar classes 1 and 2, while class 3 will be spread out over the two classes. By splitting both classes again, a third and fourth class are retrieved, and a tree structure as shown in Figure 3 is obtained. Neither the number of classes nor the encountered class-specific response probabilities will correspond to what could be expected. Hence, using only binary splits is not appropriate in this case and a ternary split, or three-class LC model, as shown in Figure 4, should be preferred. Note that this is not an LCT yet, but further splitting one of the three classes results in a tree structure.

The third configuration, shown in in Figure 2(c), contains four classes. Applying a binary split in this situation results in a child node combining classes 1 and 2 with response probabilities of 0.8 and another node combining classes 3 and 4 with response probabilities of 0.2 on the other side. Each of these combinations is split further, resulting in the tree structure shown in Figure 3 with both the expected number of classes and the appropriate conditional response probabilities.

Though these illustrative examples are somewhat artificial, they show clearly that binary splits are more appropriate in some situations than in others. Of course, in real-life applications requiring a large number of classes, it will be much more difficult to determine whether the binary split restriction is problematic. In such more complex situations, it cannot be expected that the (extended) LCT procedure will always retrieve the true classes, but the same applies to a traditional LC analysis. In general, whenever a smaller number of classes is used than indicated by the information criterion at hand, the model will be oversimplified. This is not only an inherent consequence of any tree procedure, as has been well established within the area of data mining (e.g., Kohavi and Quinlan 2002), but it also occurs when a standard LC analysis gives too many classes to be useful and the researcher prefers substantive criteria and ignores the fit measures. A nice feature of the stepwise splitting approach is that it can be applied to an LC model with any number of starting classes, where additional hidden information of interest may be picked up by the tree. Therefore, we propose a hybrid of the standard LC model and the binary LCT method, in which an initial, oversimplified but interpretable model is refined using an LCT approach. This gives a better statistical fit than the purely substantive approach, but it also allows for interpretable classes.

Various approaches can be used to decide on the number of classes of an initial LC model. If a researcher has theoretical reasons for a certain number of classes, this number can be used for the initial LC analysis. When a priori knowledge or beliefs about the number of classes is absent, we may select the number of starting classes so that they have a clear interpretation. Note that although choosing the number of starting classes based on what is substantively meaningful ignores the statistical fit of the model, model fit is still warranted since LCT picks up remaining associations (i.e., misfit) when classes are split up farther down the tree. We also present a method for choosing the number of starting classes based on the statistical fit index. More specifically, we propose choosing the number of classes in the first split based on a relative improvement in fit measure.

In principle any subsequent splits do not have to be binary also, but the focus in this article will be on an initial LC model (in other words, the root of the tree on the complete data). The initial model will pick up the most dominant associations in the data, and for any subsequent splits a deviation of the binary procedure can be supported by substantive information.

The remainder of this article is set up as follows. In section 2 we discuss the basic LC model and how it can be used to build an LCT. After that we describe the measure of relative improvement in fit that we propose to determine the split size at the root, and moreover we present a small simulation study on its performance in the situations depicted in Figure 2. In section 3 we present two empirical examples illustrating how the improvement of fit measure and substantive reasoning can be used to determine the appropriate number of classes at the first split of a tree. In section 4 we conclude the article with final remarks.

## 2. Method

### 2.1. LC Models

Let $y_{\mathrm{ij}}$ denote the response of individual i on the jth categorical variable. The responses of individual i on the full set J variables are denoted by $y_{i}$. A standard LC analysis defines a model for the probabilities of observing the various possible response patterns. Let X denote the discrete LC variable, k a particular LC, and K the number of LCs. An LC model is specified for $P(y_{i})$ as follows:

(1) $$P(y_{i})=\sum_{k=1}^{K}P(X=k)\overset{J}{\underset{j=1}{\Pi }}P(y_{\mathrm{ij}}|X=k).$$

Here, the probability of belonging to class k is represented by P(X=k), and the probabilities of all J responses conditional on belonging to class k are represented by $P(y_{\mathrm{ij}}|X=k)$. The product of the class-specific response probabilities of the J variables follows from a local independence assumption.

The model parameters are usually estimated by maximizing the likelihood through the EM algorithm (Dempster, Laird, and Rubin 1977). The log-likelihood function is as follows:

(2) $$\log L(\theta ;y)=\sum_{i=1}^{N}\log P(y_{i}),$$

where $P(y_{i})$ takes the form defined in equation (1), θ contains the model parameters P(X=k) and $P(y_{\mathrm{ij}}|X=k)$, and N denotes the total sample size.

### 2.2. Building an LCT

Building an LCT starts with the estimation of a standard one- and two-class model at the root node. If the two-class model is preferred, individuals are assigned to the two child classes having the root node as their parent. Although the current LCT model is restricted to binary splits, we show below how to decide about a possibly larger number of starting classes. Subsequently, at the next level of the tree, the child nodes become parent nodes themselves. For each parent class, one- and two-class models are estimated, and we decide whether a two-class model is preferred. If so, the cases belonging to the concerned parent class are assigned to the newly formed child classes, and the same procedure is repeated at the next level of the tree.

The model defined at a particular parent node is very similar to a standard LC model—that is, it can be formulated as follows:

(3) $$P(y_{i}|X_{\mathrm{parent}})=\sum_{k=1}^{K}P(X_{\mathrm{child}}=k|X_{\mathrm{parent}})\overset{J}{\underset{j=1}{\Pi }}P(y_{\mathrm{ij}}|X_{\mathrm{child}}=k,X_{\mathrm{parent}}),$$

where $X_{\mathrm{parent}}$ represents one of the parent classes at a particular level of the tree, and $X_{\mathrm{child}}$ represents one of the K possible newly formed child classes at the next level for the concerned parent class, where in general K equals 2. Note that each child has only one parent. Hence, $X_{\mathrm{child}}$ actually represents $X_{\mathrm{child}|\mathrm{parent}}$, but for the purpose of readability, we use the shorthand $X_{\mathrm{child}}$ throughout this article. Furthermore, $P(X_{\mathrm{child}}=k|X_{\mathrm{parent}})$ and $P(y_{\mathrm{ij}}|X_{\mathrm{child}}=k,X_{\mathrm{parent}})$ represent the class proportion and the class-specific response probabilities for child class k within the concerned parent node. In other words, as in a standard LC model we define a model for $y_{i}$, but it is now conditioned on belonging to a particular parent node.

As indicated above, if a split is accepted and new child classes are formed, observations are assigned to the newly formed classes based on their posterior class membership probabilities. More specifically, the posterior class membership probabilities for the K child nodes conditional on the parent node are obtained as follows:

(4) $$P(X_{\mathrm{child}}=k|y_{i};X_{\mathrm{parent}})=\frac{P(X_{\mathrm{child}}=k|X_{\mathrm{parent}})\overset{J}{\underset{j=1}{\Pi }}P(y_{\mathrm{ij}}|X_{\mathrm{child}}=k,X_{\mathrm{parent}})}{P(y_{i}|X_{\mathrm{parent}})}.$$

However, the actual class assignment can be done in several ways, among them using modal, random, or proportional assignment rules (Dias and Vermunt 2008). As proposed by Van der Palm et al. (2016), we use proportional class assignment in which every respondent is present at each node with a weight equal to the posterior membership probability for the node concerned.

Estimation of the LC model at the parent node $X_{\mathrm{parent}}$ involves maximizing the following weighted log-likelihood function:

(5) $$\log L(\theta ;y,X_{\mathrm{parent}})=\sum_{i=1}^{N}w_{i,X_{\mathrm{parent}}}P(y_{i}|X_{\mathrm{parent}}),$$

where $w_{i,X_{\mathrm{parent}}}$ is the weight for person i at the parent class, which equals the posterior probability of belonging to the parent class for the individual concerned. If a split is performed, the weights for the two newly formed classes at the next level are obtained as follows:

(6) $$w_{i,X_{\mathrm{child}}=1}=w_{i,X_{\mathrm{parent}}}P(X_{\mathrm{child}}=1|y_{i};X_{\mathrm{parent}})$$

(7) $$w_{i,X_{\mathrm{child}}=2}=w_{i,X_{\mathrm{parent}}}P(X_{\mathrm{child}}=2|y_{i};X_{\mathrm{parent}}).$$

In other words, a weight at a particular node equals the weight at the parent node times the posterior probability of belonging to the concerned child node conditional on belonging to the parent node. As an example, the weights $w_{i,X_{1}=2}$ used for investigating a possible split of class $X_{1}=2$ are constructed as follows:

(8) $$w_{i,X_{12}}=w_{i,X=1}P(X_{1}=2|y_{i},X=1),$$

which in turn becomes $w_{i,X=1}=P(X=1|y_{i})$. This implies

(9) $$w_{i,X_{12}}=P(X=1|y_{i})P(X_{1}=2|y_{i},X=1),$$

which shows that a weight at level 2 is in fact a product of two posterior probabilities. More details on the estimation procedure can be found in Van der Palm et al. (2016).

**Figure 5.**
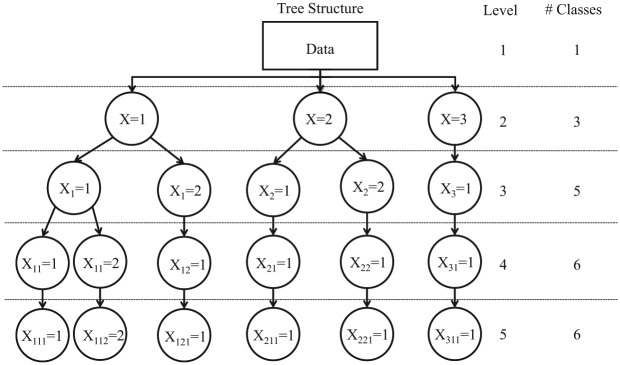
A graphical example of a latent class tree with a first split into three classes.

Construction of an LCT can be performed using standard software for LC analysis—namely, by running multiple LC models with data sets containing the appropriate case weights. After each accepted split, a new data set is constructed, and the procedure repeats itself, which is displayed in pseudocode in algorithm 1. We developed an R package that automatizes these steps and that calls an LC routine—in our case version 5.1 of the Latent GOLD program (Vermunt and Magidson 2015, 2016)—to perform the actual estimation of the LC models using the weighted data sets.^1^ This routine also provides graphical displays of the class profiles as well as the tree structure. Thus, once the tree is formed, we can use profile plots to investigate the discrepancies between classes at every split. An example of a graphical representation of an LCT can be seen in Figure 5. To prevent the structure of the tree from being affected by the fact that classes can be permuted without changing the model fit, our R routine orders the child classes within a split based on their size in descending order.

**Table:** 

**Algorithm 1.** Algorithm to Construct a Latent Class Tree
**begin** Decide on the number of classes at the first split of the tree (on the complete data) based on the relative improvement of fit measure. Make a new data set for every new class where each observation gets as a weight equal to its posterior probability for the class concerned **end** **while** Splits have been made at the previous level of the tree **do** **for** Every new class at the previous level **do** **if** A split is preferred over no split **then** Construct a new data set for each class and estimate 1 and 2 class models to decide whether a further split is needed;**end** **end** **end**

### 2.3. Statistics Used to Define the Splits

Different types of statistics can be used to determine whether a split should be accepted or rejected. Here, we use BIC (Schwarz 1978), which is defined as follows:

(10) $$\mathrm{BIC}=-2\log L(\theta ;y,X_{\mathrm{parent}})+\log(N)P,$$

where logL(.) represents the log-likelihood at the concerned parent node, N the total sample size, and P the number of parameters of the model at hand. Thus, a split is performed if at the concerned parent node BIC for the two-class model is lower than that of the one-class model. Note that using a less strict criterion (such as AIC) yields the same splits as BIC but also possible additional splits and thus a larger tree. In other words, depending on whether we wish for a smaller or a larger tree, a more conservative or a more liberal criterion may be used.

As explained in section 1, in some situations a binary split may be too much of a simplification, and we would prefer to allow for more than two classes. This is especially true for the first split of the tree, in which we pick up the most dominant features in the data. However, for this purpose, we cannot use the usual criteria such as an AIC or BIC, as this would result in using a standard LCT model again. For the decision to use more than two classes at the first split, we propose instead looking at the relative improvement of fit compared with the improvement between the one- and two-class models. When using the log-likelihood value as the fit measure, this implies assessing the increase in log-likelihood between, say, the two- and three-class model and comparing it with the increase between the one- and two-class model. More explicitly, the relative improvement between models with K and K+1 classes ($RI_{K,K+1}$) can be computed as

(11) $$RI_{K,K+1}=\frac{\log L_{K+1}-\log L_{K}}{\log L_{2}-\log L_{1}},$$

which yields a number between 0 and 1, where a small value indicates that the K-class model can be used as the first split, while a larger value indicates that the tree might improve with an additional class at the first split of the tree. Note that instead of an increase in log-likelihood, in equation (11) we may use other measures of improvement of fit, such as the decrease of BIC or AIC.

**Figure 6.**
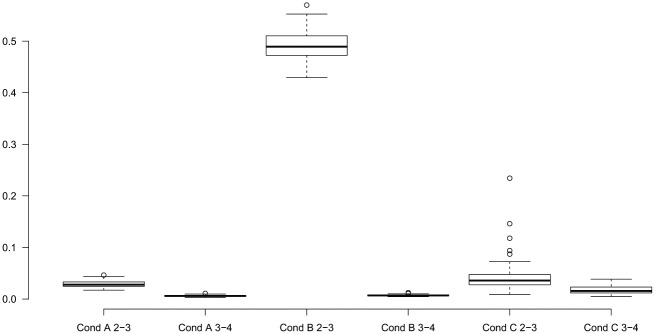
The boxplots of the improvement in fit from two to three and from three to four classes relative to the improvement from one to two classes, based on the configurations presented in Figure 2.*Note:* Cond = condition.

To get an indication of the performance of $RI_{K,K+1}$, we run a small simulation study using the three scenarios discussed in section 1 and depicted in Figure 2. For each scenario we generated 100 data sets containing 10 dichotomous response variables for 1,000 respondents and assuming equal class sizes. The results on the relative improvements from two to three classes and from three to four classes are shown via the boxplots in Figure 6.

For configuration A, binary splits suffice, as is shown by the always very low relative improvement when adding a third class. For configuration B, a ternary split is more suitable, which is confirmed by the high relative improvement in fit when increasing the classes from two to three obtained for every simulation replication. For configuration C, our measure indicates that a binary option suffices since the relative improvement was smaller than .10 for most of the simulation replications. Compared with the first configuration, the sampling fluctuation is somewhat larger in this configuration, which explains why somewhat larger values were found in a small portion of the simulation replications.

## 3. Empirical Examples

The proposed LCT methodology is illustrated by the analyses of two data sets that were previously studied using a standard LC model. The data set in the first example comes from a study by Owen and Videras (2009) and contains both a large number of respondents and a large number of variables, yielding a situation for which LCTs are well suited. For this data set, we compare the original LC solution by Owen and Videras, the first splits of a binary LCT, and an LCT with a more appropriate number of child classes at the root using our relative improvement of fit measure. The second example concerns a very large data set in terms of the number of observations from Moors and Vermunt (2007) and uses an LC model for ranking data. An LCT is well suited for this data set, as a traditional LC analysis indicates that the fit improves up to a large number of classes.

### 3.1. Social Capital

**Figure 7.**
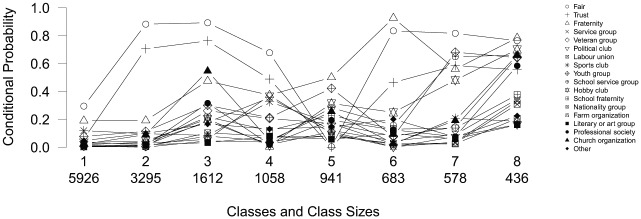
A profile plot of a standard latent class analysis on social capital.

Owen and Videras (2009:556) used the information from 14,527 respondents of several samples of the General Social Survey to construct “a typology of social capital that accounts for the different incentives that networks provide.” Social capital is a construct that is plagued by “conceptual vagueness” (Durlauf and Fafchamps 2004), and therefore Owen and Videras perform an LC analysis to grasp this concept. The data set used by Owen and Videras contains 16 dichotomous variables indicating whether respondents participate in specific types of voluntary organizations (the organizations are listed in the legend in Figure 7) and two variables indicating whether respondents agree with the statements “other people are fair” and “other people can be trusted.” Owen and Videras explain the inclusion of the latter two variables by stating that social capital is a multidimensional concept that embeds multiple manifestations of civic engagement as well as trust and fairness. Using BIC, Owen and Videras selected a model with eight classes while allowing for one local dependency—namely, between the variables fraternity and school fraternity. The eight-class original solution by Owen and Videras is displayed in Figure 7,^2^ with the size of the classes displayed on the *x*-axis.

The classes retrieved by Owen and Videras (2009) are quite difficult to interpret. Classes 1 and 2 seem to differ mainly on the variables *fair* and *trust*, while classes 2 and 3 differ on almost all variables but *fair* and *trust.* The differences between classes 1 and 3 are subsequently much harder to pinpoint, and this becomes increasingly difficult when including the other classes in the comparisons. Note furthermore that several of these classes contain small class proportion (classes 4 to 8 each contain less than 10 percent of the observations). To facilitate the interpretation of a classification of social capital, an LCT is built with this data.

**Figure 8.**
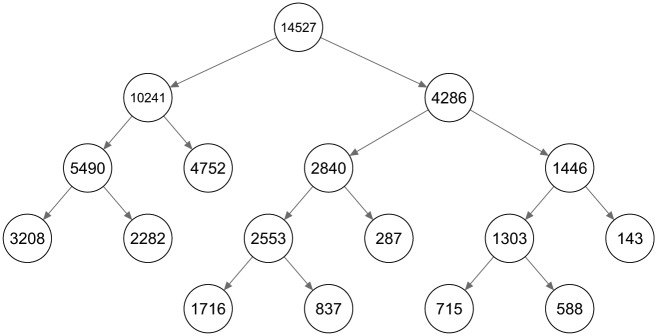
The layout of a latent class tree starting with a two-class split on the social capital data set.

The layout and class sizes^3^ of a binary LCT based on the data of Owen and Videras (2009) is shown in Figure 8. The fifth and final level of the tree consists of nine classes (every class that is not split further from a certain level is passed as it is to the next level).

**Figure 9.**
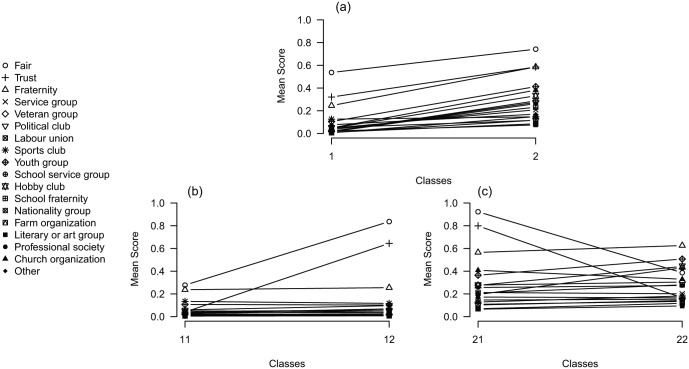
Profile plots of the first two levels of a latent class tree on social capital with only two-class splits. *Note:* Conditional response probabilities of the 18 items are shown on the *y*-axis, and different (sub)classes are shown on the *x*-axis.

The first two levels of the binary LCT can be examined more closely in their profile plots in Figure 9. Figure 9(a) shows the first split, which indicates that the probabilities on all variables^4^ are higher for class 2 than for class 1. So basically the first split divides the sample based on general social capital, wherein class 1 contains respondents with low social capital and class 2 respondents with high social capital. Figures 9(b) and 9(c) show how the classes are split further. Within each of these groups a pessimistic (classes 11 and 22) and optimistic (classes 12 and 21) social capital group seems to be present, as these groups are split mainly on the variables *fair* and *trust.* The fact that both splits at this level are mainly due to these two variables indicates that there is considerable residual association between these variables within the two classes formed at the root. Hence, a tree starting with more classes at the first split may perhaps be better suited.

**Figure 10.**
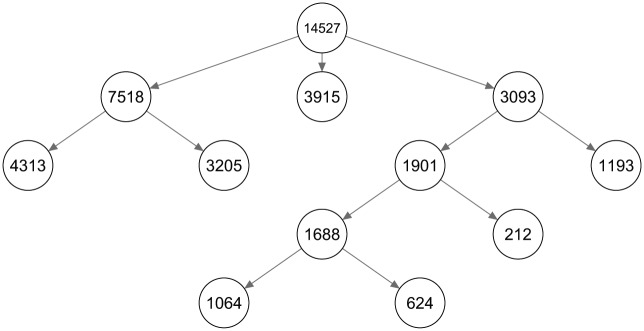
The layout of a latent class tree starting with a three-class split on the social capital data set.

To decide on the number of classes at the root of the tree, multiple standard LC models with an increasing number of classes are estimated. The fit statistics and the relative improvement of the social capital data are shown in Table 1. The relative fit improvement is about 20 percent when expanding a model from two to three classes, compared with the improvement in fit when expanding from one to two classes. Adding more classes improves the fit marginally, indicating that a root size of three classes may be used. The complete LCT obtained by starting with three classes is shown in Figure 10, with the class sizes displayed for every node of the tree. For every final node it holds that, according to BIC, a one-class model is preferred to a two-class model.

**Table 1.** Fit Statistics and Their Relative Improvement of the Social Capital Data

	logL	P	BIC	AIC	$R_{\mathrm{LL}}$	$R_{\mathrm{BIC}}$	$R_{\mathrm{AIC}}$
1	−94204	18	188581	188444			
2	−89510	37	179376	179095	1.000	1.000	1.000
3	−88501	56	177539	177115	.215	.199	.212
4	−88117	75	176952	176383	.082	.064	.078
5	−87826	94	176553	175840	.062	.043	.058
6	−87619	113	176321	175464	.044	.025	.040
7	−87425	132	176114	175113	.041	.022	.038
8	−87322	151	176090	174945	.022	.003	.018
9	−87234	170	176098	174808	.019	−.001	.015

*Note:* BIC = Bayesian information criterion; AIC = Akaike information criterion.

**Figure 11.**
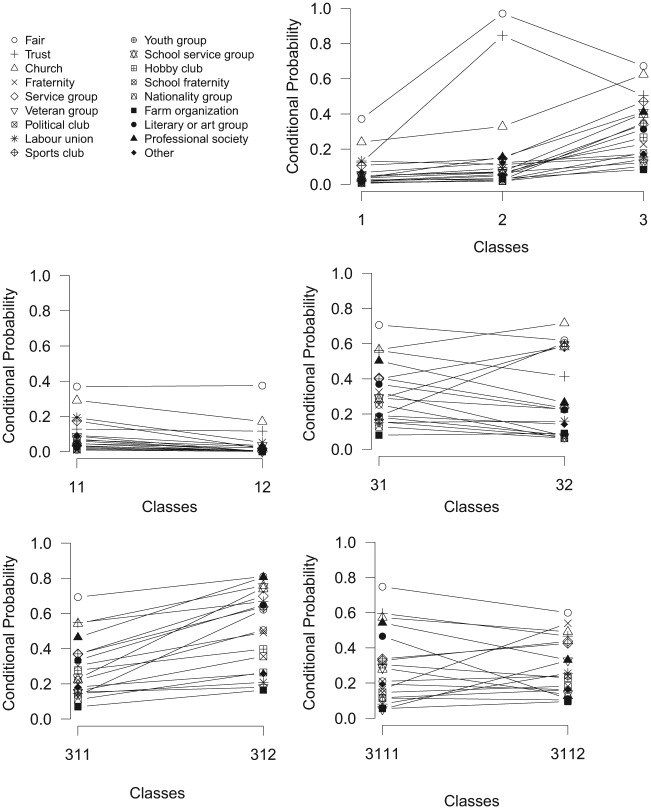
Profile plots of a latent class tree with a root of three classes on social capital.

The profile plots for the splits of the LCT with three initial classes are shown in Figure 11. At the first split, the first class has a low probability on all variables, the second class displays a low probability on participation in all voluntary organizations and very high probabilities on the variables *fair* and *trust*, and the third class displays relatively high probabilities on participation in the voluntary organizations and rather high probabilities for *fair* and *trust.* Subsequently, the first and third classes are split further, while the second is not. The first class is split in a class with low and very low probabilities on all variables, while the third class is split in two classes with preferences for different voluntary organizations (e.g., a high probability for being part of a professional organization in class 31 versus a high probability for being part of a youth group in class 32). Subsequently, class 31 is split further into classes 311 and 312, which seem to differ mainly in participation in all voluntary organizations. The final split yielding classes 3111 and 3112 results in classes that differ again in preferences for different voluntary organizations (e.g, a high probability for being part of a literary or art group in class 3111 versus a high probability for being part of a fraternity in class 3112).

The original solution of eight classes by Owen and Videras (2009) can be compared with the LCT with three initial classes. Note the resemblance between the first classes of LCT and the standard LC model. The relation between the fully binary LCT and standard LC analysis solutions is less clear, though there are similarities. For instance, LCT class 21 is rather similar to class 2 in standard LC analysis. Similarities in the results of the LCT and standard LC analysis are expected, though the goal of an LCT is not to resemble the standard LC analysis result. A great advantage of the LCT is that the classes can be interpreted stepwise—first the classes at the first level of the tree can be interpreted, and subsequently so can the classes at lower levels. Moreover, LCT offers the possibility of making a decision about the number of classes based on substantive reasons. Hence, splits at lower levels that are of no substantive interest can be ignored. For instance, the distinction between classes 11 and 12, which differ mainly in the degree of low participation in voluntary groups may be of less interest, as they reflect subtle quantitative differences rather than qualitative differences. In such a case, class 1 can be used in the final classification instead of classes 11 and 12.

### 3.2. (Post)Materialism

The study by Moors and Vermunt (2007) used the answers of 21,468 respondents participating in the 1990 European Values Survey on three questions meant to validate the measurement of (post)materialism as proposed by Inglehart (1971). Each item contained the four aims of a country, and respondents were to determine which aim should have the highest priority and which one should have the second highest priority in their opinion. The response options of the three items can be seen in Table 2.

**Table 2.** Indicators for the Latent Class Discrete Choice Model

Item A	Item B	Item C
Maintaining a high level of economic growth	Maintaining order in the nation	Maintaining a stable economy
Making sure the country has strong defense forces	Giving people more say in important government decisions	Progress toward a less impersonal and more humane society
Seeing that people have more to say about how things are done at their jobs and in their communities	Fighting rising prices	Progress toward a society in which ideas count more than money
Trying to make our cities and countryside more beautiful	Protecting freedom of speech	Fighting against crime

Moors and Vermunt (2007) used an LC discrete choice model for their study, as every respondent gave two ranked responses per item. An LC discrete choice model is quite similar to a traditional LC model as depicted in equation (1). For response pattern s, with the first and second response on an item denoted by $a_{1}s$ and $a_{2}s$, respectively, a discrete choice model has the form of

(12) $$P(y_{s})=\sum_{k=1}^{K}P(X=k)\overset{J}{\underset{j=1}{\Pi }}P(y_{1j}=a_{1s},y_{2j}=a_{2s}|X=k).$$

With an LCT approach this model become

(13) $$P(y_{s}|X_{\mathrm{parent}})=\sum_{k=1}^{K}P(X_{\mathrm{child}}=k|X_{\mathrm{parent}})\overset{J}{\underset{j=1}{\Pi }}P(y_{1j}=a_{1s},y_{2j}=a_{2s}|X_{\mathrm{child}}=k,X_{\mathrm{parent}}).$$

Within a discrete choice framework, the choice probabilities are parameterized in terms of the utilities of the alternatives. In our case, for the first item, this implies that

(14) $$P(y_{11}=a_{1s},y_{21}=a_{2s}|X_{\mathrm{child}}=k,X_{\mathrm{parent}})=\frac{\tau_{a_{1}k}}{\sum_{a=1}^{4}\tau_{\mathrm{ak}}}\frac{\tau_{a_{2}k}}{\sum_{a\neq a_{1}}^{4}\tau_{\mathrm{ak}}}.$$

A higher value of $\tau_{\mathrm{ak}}$ indicates a higher probability that someone belonging to class k selects alternative a. Two important differences with a standard LC model are that the utilities are assumed to be equal between the first and second choices and that it should be taken into account that with the first and second choices a ranking task cannot be the same, which is why the summation for the second choice is over the nonselected alternatives ($a\neq a_{1}$). As is usually done, we use log-transformed utilities, which are logit coefficients—that is,

(15) $$\log\tau_{\mathrm{ak}}=\beta_{\mathrm{ak}}.$$

For identification, effects coding is used, implying that the $\beta_{\mathrm{ak}}$ sums to 0 within LC k. These parameters can be interpreted as follows: A positive value of $\beta_{\mathrm{ak}}$ indicates a more attractive alternative a for someone belonging to the class k, while the reverse applies to negative values.

The fit statistics obtained when estimating LC discrete choice models with 1 to 10 classes, as well as the corresponding relative fit improvement, are reported in Table 3. As can be seen, the BIC and AIC values keep decreasing for 10 classes, indicating that a large number of classes should be selected based on the measures. However, the relative improvement of fit decreases rather quickly and seems to become rather small after four classes. Based on this measure, an LCT model with four starting classes thus seems to be suited for this data set.

**Table 3.** Fit Statistics and Their Relative Improvement of the Discrete Choice Data

	logL	P	BIC	AIC	$R_{\mathrm{LL}}$	$R_{\mathrm{BIC}}$	$R_{\mathrm{AIC}}$
1	−98236	9	196557	196489			
2	−95154	19	190490	190347	1.00	1.00	1.00
3	−94389	29	189056	188837	.25	.24	.25
4	−93965	39	188304	188009	.14	.12	.13
5	−93796	49	188060	187689	.05	.04	.05
6	−93678	59	187920	187474	.04	.02	.04
7	−93596	69	187853	187331	.03	.01	.02
8	−93531	79	187818	187220	.02	.01	.02
9	−93465	89	187782	187109	.02	.01	.02
10	−93416	99	187779	187030	.02	.00	.01

*Note:* BIC = Bayesian information criterion; AIC = Akaike information criterion.

**Figure 12.**
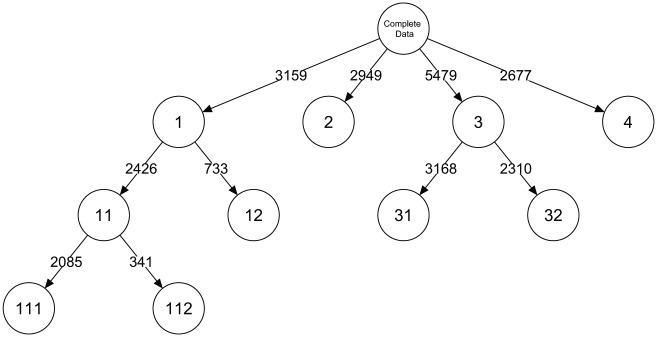
Layout and class sizes of the latent class tree based on the discrete choice data on (post)materialism.

In addition to the relative improvement of fit, other (substantive) considerations can be used to decide on the number of classes at the first split of the tree. This is also what Moors and Vermunt (2007) did in the original study. They compared the two- to five-class models and concluded that four classes could be identified in which at least one item from each set is related to a particular LC. Such substantive reasoning also can guide a decision about the number of classes, but with the LCT approach these classes can be further explored. Out of the four initial classes, two are split based on BIC, and at the final level there is one more split. This yields a total number of seven classes at the final level of the tree, as shown in Figure 12.

The estimated utilities are reported in Table 4. For the first class at the first level of the tree, it can be seen that the high utilities for the first response option of every item (namely, the issues “Maintaining a high level of economic growth,”“Maintaining order in the nation,” and “A stable economy”) shape the first class. These economic and “maintaining order” issues led Moors and Vermunt (2007) to interpret this class as a “conservative” elite class, which stresses issues of macroeconomic and socioeconomic order. For the second class the response options “Strong defense forces,”“Fighting rising prices,” and “Fighting against crime” cluster together. These issues have been interpreted as “typical” concerns of the lower class. The third class favors the more postmaterialistic response options “More say at work,”“More say in government decisions,” and “More humane society.” This class is therefore also interpreted as a postmaterialist class. The fourth and final class combines postmaterialistic and economic issues—namely, “Economic growth,”“More say in government issues,” and “A stable economy.” This is interpreted as a more democratic but also macroeconomic class.

**Table 4.** Logits of the Latent Class Discrete Choice Models

Level of the Tree	1				2				3	
Classes	1	2	3	4	11	12	31	32	111	112
*Set A*										
Economic growth	1.590	.217	.302	2.075	1.571	1.750	.438	.045	1.452	1.800
Strong defense forces	−.992	−.797	−2.178	−1.526	−1.525	−.609	−2.255	−2.123	−1.311	−1.813
More say at work	.009	.561	1.662	.440	.456	−.514	1.490	2.088	.667	.209
Beautiful cities	−.606	.019	.213	−.989	−.502	−.626	.326	−.010	−.808	−.196
*Set B*										
Maintaining order	1.678	.160	−.500	−.652	1.796	1.581	−.299	−.962	1.983	1.685
More say for people	−.924	−.334	.774	.617	−.839	−.996	.476	1.532	−.807	−.852
Fight rising prices	−.521	.470	−.893	.024	−.886	−.154	−1.183	−.618	−.696	−1.292
Freedom of speech	−.233	−.297	.619	.010	−.071	−.431	1.005	.048	−.480	.460
*Set C*										
Stable economy	1.467	.050	−.591	1.638	1.356	1.619	−.663	−.488	1.367	1.353
Humane society	−.484	−.206	1.050	−.223	−.366	−.648	.918	1.314	−.222	−.577
Ideas count	−1.415	−.658	.112	−1.102	−1.365	−1.476	.188	−.014	−1.465	−1.234
Fight against crime	.432	.814	−.570	−.313	.375	.504	−.443	−.813	.319	.459

These four classes at the first level are the same as those identified by Moors and Vermunt (2007) using a traditional LC analysis. However, the tree extension allows for a more detailed picture regarding the more subtle variation within these four classes. The first thing that stands out is that only classes 1 and 3 are split into subclasses. The first, so-called conservative elite class splits into two classes that differ mainly in how much they like or dislike “More say at work” on the first item and how much they like or dislike “Strong defense forces” on the first item and “Fighting rising prices” on the second item. The third class at the first level, labeled the postmaterialist class, is split into two classes that mainly differ in the importance attributed to “Protecting freedom of speech” and “Giving people more say in important government decisions.” Hence, we can distinguish two groups here that differ in their preference for the postmaterialistic aspects. At the final level of the tree, the so-called conservative elite class that focused mainly on economic growth is split further. This split is based mainly on the difference between the first and second item, wherein class 111 has a stronger preference for the options “Strong defense forces” and “More say at work” on the first item and the option “Fighting rising prices” on the second item, and class 112 has a stronger preference for the option “Beautiful cities and countryside” on the first item and “Protecting freedom of speech” on the second item.

To summarize, the tree starts with four branches that correspond with the four classes of the original solution by Moors and Vermunt (2007) and subsequently yields five subclasses spread over two branches. The final result at the lowest level of the tree consists of seven classes, but it is possible to decide on the most interesting number of classes of LCT with substantive reasoning. For instance, if for a particular study specific clusters of an elite class but not a division of the postmaterialistic class are of interest, classes 31 and 32 can be replaced by class 3.

## 4. Discussion

The LCT models approach discussed in this article provides an alternative approach to LC analysis, in which a stepwise procedure is used to build a meaningful cluster model for the data set at hand. LCT models are especially useful when standard LC models would yield a large number of classes with mutual differences that are difficult to interpret. Because the restriction of the current LCT to binary splits can be problematic, we proposed a modification allowing for a larger number of child classes at the root of LCT. We introduced a relative improvement of fit measure to decide about the number of classes, which turned out to work well in our small simulation study. We illustrated the new approach using two empirical examples, in which the relative improvement of fit measure indicated that we should use three and four starting classes, respectively. For the first example, we also compared trees starting with two and three classes and showed that the latter yielded a much more easily interpretable clustering.

Although the option of using nonbinary splits in the current article has been applied only to the first split of LCT, in principle it could also be used at the next levels of a tree. For instance, in the first example on social capital, both class 1 and class 3 could be split into more than two classes. Based on BIC, this would be three and six classes, respectively. Rather than using BIC, it may be possible to adapt our measure of relative improvement for this purpose—for instance, by comparing the improvement of fit with the one at the first split or with the one within the branch at hand. Because the number of classes at the splits can strongly affect the outcome of an LCT analysis, we recommend deciding this separately for every split, starting with the first split. Note that at lower levels of the tree more substantive information about the branch is already available and can be used to guide the decision regarding the number of subclasses.

The LCT models described in this article are somewhat similar to the LC factor models proposed by Magidson and Vermunt (2001). For example, a tree with binary splits at the first and second level resembles an LC factor model with two dichotomous latent factors. However, in LC factor models, the number of factors as well as the number of categories of the factors can be increased. Although this is similar to increasing the number of subclasses in a split as discussed in this article, an important difference is that the multiple classes corresponding to the same factor are restricted to be ordered. It may be worth investigating whether such an approach—in which the number of classes is increased but at the same time the classes are restricted to be ordered—is useful in the context of LCT models as well. For instance, in our example on social capital, we may wish to force the splits at the first and second levels to represent different dimensions, using possibly more than two classes. In such a case, it would make sense to apply an LC factor approach at these splits of LCT.

In this article, we used BIC to decide whether to stop the splitting process of the classes. Although BIC has been shown to perform well for standard LC analysis (Nylund, Asparouhov, and Muthén 2007), various other model selection criteria such as the integrated classification likelihood (Biernacki, Celeux, and Govaert 2000) are available. Their strictness influences the probability of starting a new branch within an LCT, implying that the choice for the decision criterion can affect the bottom of the tree significantly. Whereas we used the standard maximum likelihood method for the estimation of the submodels forming an LCT, it may be worth considering other estimation procedures, such as the recently proposed minimum ϕ-divergence estimation method (Felipe, Miranda, and Pardo 2015).

Summarizing this information, we can see that various options are available for deciding on the size of the splits of an LCT. In a purely exploratory analysis, the proposed relative improvement of fit measure seems to be a useful tool for deciding about the number of starting classes, while in other situations we may wish to base this decision on content information. The form of the tree and thus the composition of the classes will therefore be subject to the available information and requirements of the research question at hand. There are many ways to derive a clustering from a data set, and it is best to assume that there is no particular method that is correct in all situations (Hennig 2015). In other words, we do not want to claim that the LCT approach will always yield the best results or the true clusters, but this is often also unlikely for a standard LC analysis. In practice, a researcher may start with a standard LC analysis and switch to our LCT approach when encountering difficulties in deciding about the number of classes or interpreting the differences between a possibly large number of classes.

## Appendix

**Table A1.** Conditional Probabilities and Class Sizes of the LC Analysis on Social Capital

	C1	C2	C3	C4	C5	C6	C7	C8
Fair	.29	.88	.89	.68	.00	.84	.82	.76
Trust	.19	.19	.48	.37	.50	.93	.56	.79
Fraternity	.06	.71	.76	.49	.00	.46	.59	.56
Service group	.02	.03	.18	.35	.12	.05	.09	.34
Veteran group	.01	.00	.26	.21	.17	.08	.14	.65
Political club	.03	.03	.06	.37	.10	.00	.08	.16
Labor union	.00	.00	.10	.08	.08	.03	.03	.31
Sports club	.12	.10	.08	.33	.17	.02	.21	.19
Youth group	.08	.11	.30	.21	.42	.08	.68	.64
School service	.01	.00	.05	.04	.28	.16	.65	.66
Hobby club	.03	.04	.20	.01	.31	.25	.48	.70
School fraternity	.02	.04	.22	.12	.23	.13	.14	.38
Nationality group	.00	.01	.19	.02	.11	.00	.04	.33
Farm organization	.01	.01	.08	.04	.08	.02	.02	.21
Literary or art group	.01	.02	.03	.08	.06	.10	.06	.16
Professional society	.01	.01	.32	.02	.19	.12	.08	.58
Church organization	.02	.09	.55	.05	.26	.03	.19	.66
Other	.05	.10	.17	.13	.15	.20	.07	.22
Class sizes	.41	.23	.11	.07	.06	.05	.04	.03

**Table A2.** Conditional Probabilities and Class Sizes of the LCT Starting with a Split of Two Classes on Social Capital

	1	2	11	12	21	22	111	112	211	212	221	222	2111	2112	2211	2212
Fair	.54	.74	.28	.84	.92	.39	.29	.26	.92	.97	.37	.54	.90	.95	.42	.30
Trust	.32	.59	.04	.64	.80	.16	.05	.03	.79	.88	.15	.28	.78	.82	.16	.14
Fraternity	.05	.21	.04	.06	.21	.20	.06	.00	.19	.43	.19	.34	.25	.05	.09	.30
Service group	.02	.29	.02	.02	.28	.30	.03	.00	.23	.66	.27	.66	.27	.16	.20	.34
Veteran group	.05	.12	.05	.06	.10	.14	.08	.00	.10	.11	.13	.21	.13	.05	.07	.21
Political club	.01	.12	.01	.01	.11	.13	.01	.00	.09	.30	.11	.37	.10	.06	.07	.15
Labor union	.13	.15	.13	.12	.13	.18	.19	.05	.13	.14	.18	.23	.13	.11	.18	.18
Sports club	.10	.41	.11	.10	.36	.51	.17	.01	.34	.54	.48	.77	.29	.46	.55	.39
Youth group	.02	.27	.03	.01	.19	.43	.05	.00	.17	.42	.39	.77	.06	.40	.62	.12
School service	.05	.33	.05	.04	.28	.44	.07	.03	.24	.57	.40	.82	.13	.48	.58	.18
Hobby club	.04	.23	.03	.04	.21	.28	.06	.00	.20	.31	.26	.49	.21	.16	.23	.30
School fraternity	.01	.15	.00	.01	.15	.15	.01	.00	.11	.43	.13	.40	.15	.04	.07	.20
Nationality group	.01	.09	.01	.01	.07	.12	.02	.00	.06	.17	.10	.31	.07	.04	.07	.13
Farm organization	.02	.08	.02	.03	.07	.09	.03	.01	.06	.10	.08	.26	.06	.07	.08	.08
Literary or art group	.01	.26	.02	.01	.26	.27	.02	.00	.22	.61	.23	.64	.23	.18	.20	.27
Professional society	.05	.38	.03	.07	.41	.33	.04	.02	.37	.77	.29	.70	.41	.28	.23	.36
Church organization	.25	.59	.24	.26	.57	.63	.29	.16	.55	.69	.60	.88	.49	.68	.69	.48
Other	.08	.17	.06	.10	.17	.17	.09	.02	.17	.19	.15	.29	.18	.15	.13	.18
Class sizes	.70	.30	.38	.33	.20	.10	.22	.16	.18	.02	.09	.01	.12	.06	.05	.04

**Table A3.** Conditional Probabilities and Class Sizes of the LCT Starting with a Split of Three Classes on Social Capital

	1	2	3	11	12	31	32	311	312	3111	3112
Fair	.37	.97	.67	.37	.37	.71	.62	.69	.81	.75	.60
Trust	.12	.84	.50	.13	.12	.56	.41	.55	.67	.60	.47
Fraternity	.04	.09	.23	.07	.00	.32	.08	.30	.49	.17	.54
Service group	.02	.06	.34	.03	.00	.41	.23	.37	.70	.33	.44
Veteran group	.05	.07	.12	.09	.00	.16	.07	.15	.18	.05	.33
Political club	.01	.02	.14	.01	.00	.18	.08	.16	.36	.15	.18
Labor union	.13	.11	.16	.19	.05	.15	.16	.15	.21	.08	.26
Sports club	.11	.15	.47	.17	.02	.40	.58	.37	.64	.34	.43
Youth group	.03	.03	.35	.05	.00	.19	.60	.13	.62	.12	.16
School service	.05	.07	.40	.07	.03	.29	.59	.23	.75	.28	.13
Hobby club	.04	.07	.27	.06	.00	.29	.23	.28	.40	.31	.23
School fraternity	.01	.03	.18	.01	.00	.25	.06	.22	.50	.21	.24
Nationality group	.01	.02	.10	.02	.00	.13	.06	.11	.26	.11	.10
Farm organization	.02	.03	.08	.03	.01	.08	.09	.07	.16	.05	.09
Literary or art group	.02	.05	.31	.02	.00	.37	.22	.33	.65	.47	.11
Professional society	.03	.15	.41	.04	.02	.50	.27	.46	.81	.54	.33
Church organization	.24	.33	.62	.29	.17	.57	.72	.54	.76	.57	.49
Other	.07	.12	.17	.09	.03	.19	.14	.18	.26	.19	.16
Class sizes	.52	.27	.21	.30	.22	.13	.08	.12	.01	.07	.04

## Notes

1. A similar, fully automated estimation procedure for latent class trees will be implemented in version 6.0 of Latent GOLD.

2. The exact conditional probabilities of the latent class model and latent class trees on social capital can be found in the appendix.

3. Every split should sum up to the class size of its parent node. However, because the allocation is carried out on the basis of the posterior probabilities, the class sizes are not integers. For convenience, these numbers have been rounded, which causes slight deviations where the sum of two child nodes does not exactly add up to the parent node.

4. The exact probabilities can be found in the appendix.
